# Supplementary material for: Evidence supported by Mendelian randomization: impact on inflammatory factors in knee osteoarthritis
Source: Front Med (Lausanne). 2024 May 28;11:1382836. doi: 10.3389/fmed.2024.1382836 (PMC11165061; doi:10.3389/fmed.2024.1382836)
Supplement: Supplementary file 1 [file Data_Sheet_1.ZIP › data/GCST90274839_5e6/again/839.forest.pdf]

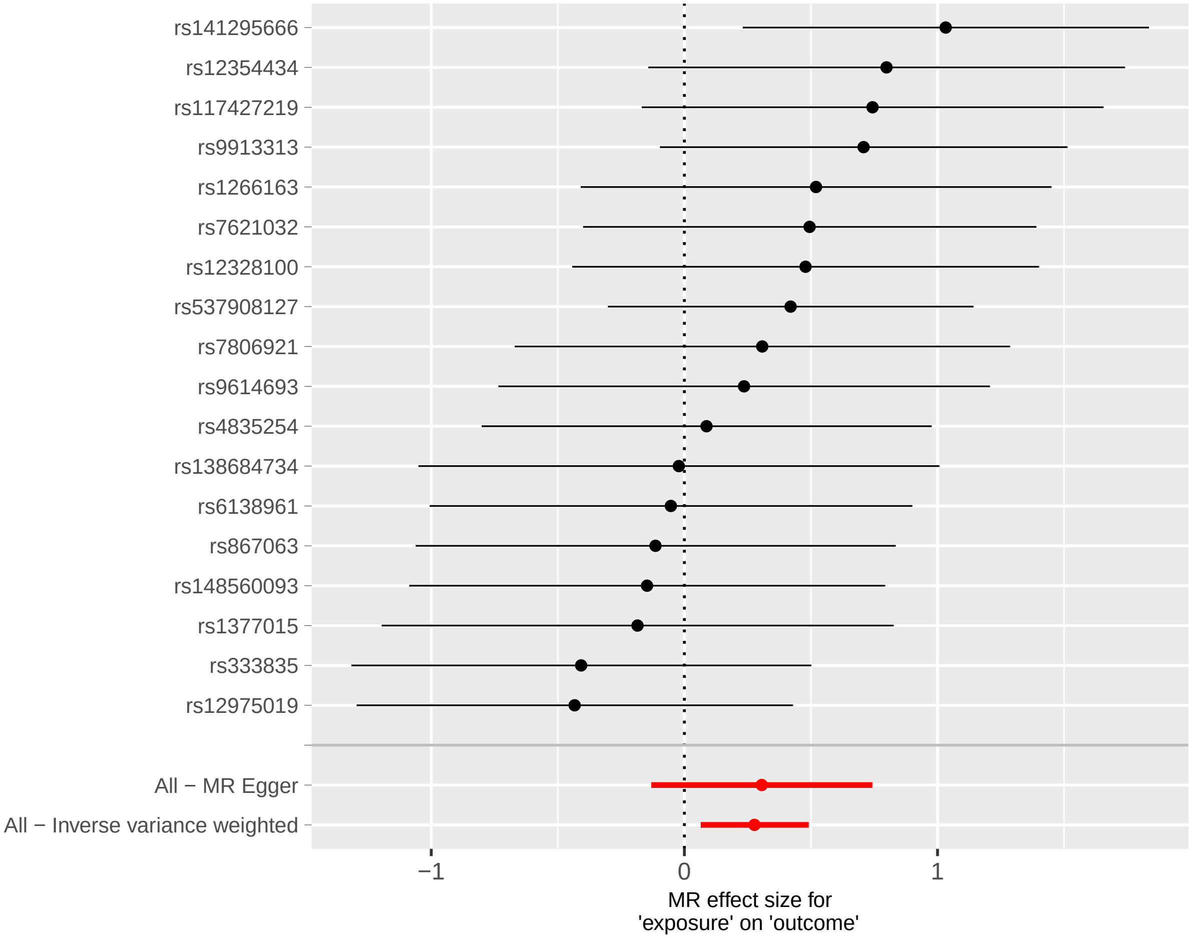
rs141295666
rs12354434
rs117427219
rs9913313
rs1266163
rs7621032
rs12328100
rs537908127
rs7806921
rs9614693
rs4835254
rs138684734
rs6138961
rs867063
rs148560093
rs1377015
rs333835
rs12975019
All − MR Egger
All − Inverse variance weighted
−1
0
1
MR effect size for
'exposure' on 'outcome'
